# Enhancing the Extinction Efficiency and Plasmonic Response of Bimetallic Nanoparticles of Au-Ag in Robust Thin Film Sensing Platforms

**DOI:** 10.3390/s23239618

**Published:** 2023-12-04

**Authors:** Diana I. Meira, Marco S. Rodrigues, Joel Borges, Filipe Vaz

**Affiliations:** 1 Physics Center of Minho and Porto Universities (CF-UM-UP), University of Minho, Campus de Azurém, 4800-058 Guimarães, Portugal; dianaisabelameira@gmail.com (D.I.M.);; 2 LaPMET–Laboratory of Physics for Materials and Emergent Technologies, University of Minho, Campus de Gualtar, 4710-057 Braga, Portugal

**Keywords:** plasmonics, Localized Surface Plasmon Resonance (LSPR), AuAg-TiO_2_ thin film, Au-Ag nanoparticle, refractive index sensitivity (RIS)

## Abstract

The extinction efficiency of noble metal nanoparticles (NPs), namely gold (Au) and silver (Ag), are dependent on their size and surrounding dielectric. Exploiting the Localized Surface Plasmon Resonance (LSPR) phenomenon, the composition and structure of the NPs might be tailored to achieve a configuration that optimizes their response (sensitivity) to environmental changes. This can be done by preparing a bimetallic system, benefiting from the chemical stability of Au NPs and the higher scattering efficiency of Ag NPs. To enhance the LSPR sensing robustness, incorporating solid supports in the form of nanocomposite thin films is a suitable alternative. In this context, the NPs composed of gold (Au), silver (Ag), and their mixture in bimetallic Au-Ag NPs, were grown in a titanium dioxide (TiO_2_) matrix using reactive DC magnetron sputtering. Thermal treatment at different temperatures (up to 700 °C) tuned the LSPR response of the films and, consequently, their sensitivity. Notably, the bimetallic film with Au/Ag atomic ratio 1 exhibited the highest refractive index sensitivity (RIS), with a value of 181 nm/RIU, almost one order of magnitude higher than monometallic Au-TiO_2_. The nanostructural analysis revealed a wide NP size distribution of bimetallic NPs with an average size of 31 nm, covering about 20% of the overall surface area. These findings underscore the significant potential of bimetallic film systems, namely AuAg-TiO_2_, in LSPR sensing enhancement.

## 1. Introduction

Several nanoscience areas (e.g., materials, physical, chemical, and biological) take advantage of the Localized Surface Plasmon Resonance (LSPR) phenomenon by exploring the optical properties of metallic nanostructures [1,2,3]. The LSPR phenomenon can be interpreted as an interaction between an incident electromagnetic field and the free electrons of metallic nanoparticles (NPs), resulting in a collective oscillation of the electron cloud with a certain resonance frequency [4,5,6,7]. These plasmon excitations are non-propagating, i.e., are highly confined to the metal nanostructure-dielectric interface. The resultant light–matter interaction gives rise to several distinct phenomena: (i) strong extinction (LSPR) bands, due to absorption and scattering, when the wavelength of the incident light is about one order of magnitude higher than the NP size, and (ii) enhancement of the electromagnetic field near the metallic surface [8,9,10]. The LSPR band position, intensity, and width can be tuned by metal composition, geometrical characteristics (size, shape, and interparticle distance) of the nanostructures, and surrounding conditions (e.g., temperature, pressure, and refractive index (RI)) [6,11,12,13,14].

**Figure 1:**
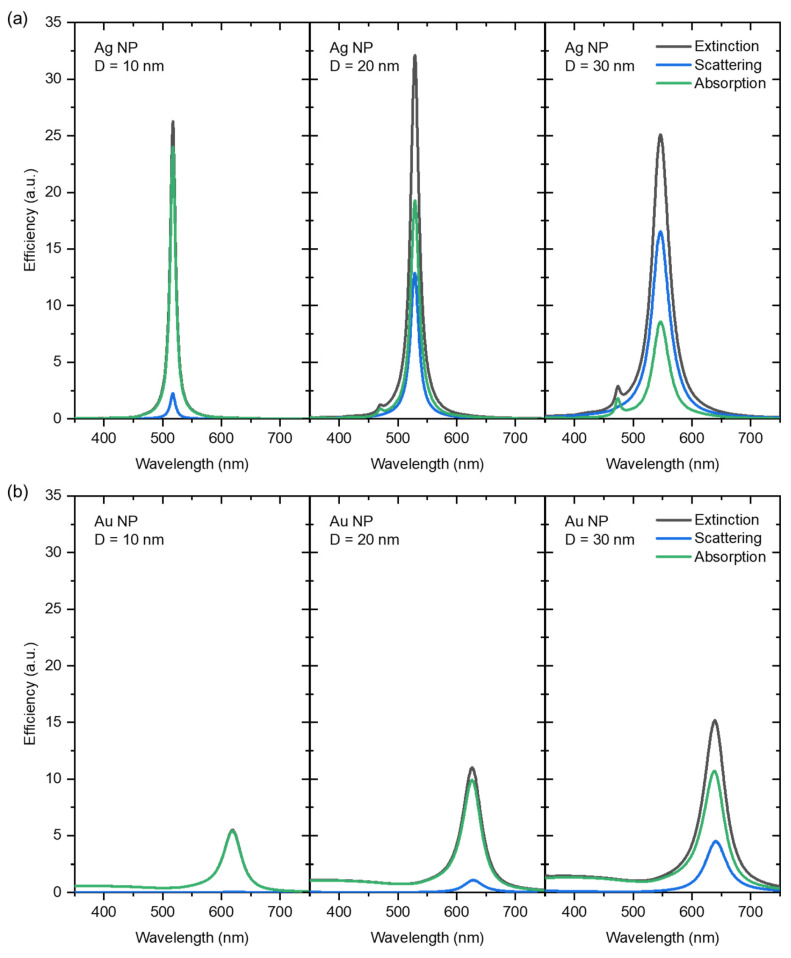
Calculated extinction, scattering, and absorption efficiencies of a spherical nanoparticle with variable size D (D = 10, 20, 30 nm), composed of (**a**) Ag and (**b**) Au, surrounded by a dielectric medium with a constant refractive index of 2.3 (simulating a TiO_2_ matrix). Calculations were performed using a software from Nanohub.org [22].

The most commonly studied noble metal nanostructures are Au and Ag NPs since their resonance frequency falls into the visible region of the electromagnetic spectrum [15,16]. In plasmonics applications, the Au NPs present some pertinent features: inert nature and stability, relatively simple synthesis bioconjugation, as well as, bio-inertness, biocompatibility, and poor toxicity [17,18]. In turn, the Ag NPs reveal higher extinction cross-sections (i.e., higher absorption and scattering efficiencies), yet for higher energies (lower wavelengths) [19,20]. In addition, it also exhibits a higher inherent optical loss compared to the Au NPs [21]. To demonstrate these features, Figure 1 depicts the calculated spectra of extinction, scattering, and absorption efficiencies for the Au and Ag NPs with the same size and surrounding dielectric medium. The efficiency spectra were calculated using a software from Nanohub.org (v1.03UQ) [22], considering spherical shapes and three different diameters (10, 20, and 30 nm), embedded in a dielectric medium with a refractive index of 2.3 (close to the anatase phase of titanium dioxide (TiO_2_), as previously calculated by the authors [23]).

As depicted in Figure 1, the absorption and scattering efficiencies can be tuned by the NP size. For instance, if the NP is smaller than 20 nm, the predominant process is absorption. An increase in NP size leads to a scattering efficiency enhancement [5,24,25,26]. Comparing both monometallic counterparts at the same conditions, the Ag NPs are more efficient in scattering light at the resonance condition and, therefore, will exhibit enhanced sensitivity factors, i.e., higher responsiveness to environmental changes [27].

**Figure 2:**
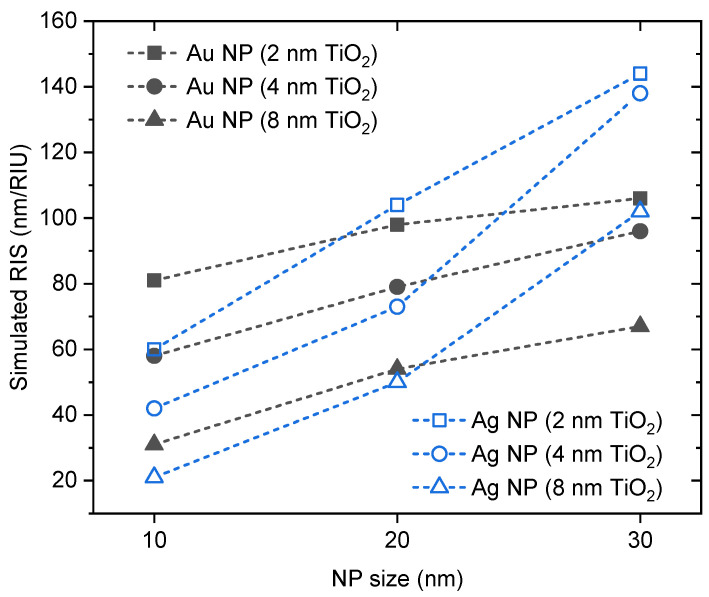
Simulated refractive index sensitivity (RIS) of a spherical nanoparticle (D = 10, 20, 30 nm) surrounded by a TiO_2_ finite layer (d = 2, 4, 8 nm), immersed into two environments with different refractive index units, η_1_ (RIU) = 1.333 and η_2_ (RIU) = 1.381 (Δη = 0.048) [22]. RIS was calculated by the difference in the wavelength of extinction maxima, divided by the difference in refractive index units, according to RIS = (λ_2_ − λ_1_)/Δη.

In Figure 2, the refractive index sensitivity (RIS) was simulated for both the Au and Ag NPs covered with a TiO_2_ cap layer, considering different NPs sizes and layer thicknesses. Considering the same layer thicknesses, the sensing ability of both the metallic NPs is enhanced with the increasing NPs size. Moreover, Figure 2 also highlights the significant influence of the surrounding media on the NP’s sensing ability. Variations in the refractive index of the NP surrounding medium will lead to a measurable resonance band shift. However, this shift only occurs if the refractive index variation takes place in the sensing volume contained within the plasmon’s electric field decay length. The plasmon field decay decreases exponentially with the distance from the NP, limited between a length range of 10 to 30 nm, depending on the NP size and shape [28,29]. Hence, increasing the TiO_2_ layer around the NP will lead to a decrease in the intensity of the LSPR extinction band and, consequently, in the sensing ability [30].

Typically, the noble metal monometallic NPs have been recognized for a broad range of sensing applications. These NPs are frequently commercialized as colloids, and an example of their use is the Lateral Flow Immunoassay (LFIA) platforms (e.g., the COVID test [31]). However, the synergistic combination of the Au and Ag NPs in bimetallic systems may offer several advantages in different dimensions (e.g., economic, and functional aspects in plasmonic sensing technology), compared to their monometallic counterparts [27,32,33]. Incorporating the Au NPs in bimetallic systems improves the chemical and thermal stability of the Ag NPs, preventing oxidation and enabling biocompatibility [21,34,35]. In turn, the Ag NPs can enhance and tune the plasmonic properties, while being reasonably priced [36]. All of these characteristics and properties have enabled the development of more effective plasmonic (e.g., LSPR [15,37,38,39,40,41], SERS (surface-enhanced Raman spectroscopy) [42,43], and SPCE (surface plasmon-coupled emission) [44,45,46]) sensing applications. For instance, Alkhalayfeh et al. [47] reported a significant enhancement of broadband light absorption in polymer solar cells by up to 69.6% due to the LSPR effect of the incorporated combined Au-Ag NPs. Regarding a biosensing application example, Zheng et al. [48] reported a novel aptasensor for ochratoxin-A detection based on enhanced electromagnetic fields (near-field) caused by the plasmonic coupling behavior of the bimetallic Au-Ag Janus NPs. Furthermore, Costa et al. [49] prepared bimetallic Au-Ag NPs dispersed in a TiO_2_ matrix. In that work, the thin films have been reported as potentially efficient LSPR-based sensing platforms since they were able to reproduce significant changes in the LSPR band’s parameters (i.e., wavelength shift and LSPR band central moments) upon different surrounding media.

In the present work, the sensing ability of the Au-TiO_2_, Ag-TiO_2,_ and bimetallic AuAg-TiO_2_ nanocomposite thin films was analyzed to develop an LSPR-sensing platform with optimal sensitivity. For that, nanocomposite thin films were deposited by reactive DC magnetron sputtering, using a Ti target with varying amounts of the Au and/or Ag pellets to obtain different noble metal ratios (from Au and Ag to Au-Ag in different ratios) throughout the TiO_2_ dielectric matrix. Following the deposition, the nanocomposite thin films were thermally annealed at different temperatures. This treatment favors the growth and coalescence of NPs and, consequently, develops an enhanced plasmonic response. To identify the LSPR response of the thin films and assess their sensitivity (refractive index sensitivity, RIS), optical (transmittance) spectra measurements were conducted. Further, the LSPR sensing experiments were performed using deionized water and a model solution of sucrose (30% *w*/*w*), exhibiting different RI. The surface morphology and nanostructure of the nanocomposite thin film that revealed the highest RIS were further investigated, correlating both Scanning Electron Microscopy (SEM) and Atomic Force Microscopy (AFM) analysis.

## 2. Materials and Methods

### 2.1. Reactive DC Magnetron Sputtering Deposition

Nanocomposite thin films composed of Au NPs, Ag NPs, and Au-Ag NPs dispersed throughout a TiO_2_ dielectric matrix were deposited onto quartz substrates (SiO_2_) (quality JS1from Neyco Vacuum and Materials, Vanves, France). The substrates were placed on a hexagonal sample holder centered on the high-vacuum sputtering chamber, 70 mm far from the target. Before the deposition, the substrates were treated and activated, using an in situ etching process with the Ar plasma using a pulsed DC power supply (model TruPlasma DC 4005 from Hüttinger Elektronik, Nuremberg, Germany), while the sample holder rotated at 5.5 rpm speed. The sputtering target was composed of three-quarters of a noble metal disk (total area, 12 mm^2^) symmetrically placed in the erosion track of a rectangular titanium (Ti) target (200 × 100 × 6 mm^3^, 99.99% purity) from Testbourne Ltd., Hampshire, England. The noble metal pellet area varied between 12 mm^2^ of Au, 12 mm^2^ of Ag, and mixtures of Au and Ag (8 mm^2^ of Au + 4 mm^2^ of Ag and 4 mm^2^ of Au + 8 mm^2^ of Ag). In a plasma composed of Ar (working gas, 3.8 × 10^−1^ Pa) and O_2_ (reactive gas, 3.6 × 10^−2^ Pa), the target was sputtered for 8 min, aiming to deposit a nanocomposite thin film with a thickness of approximately 50 nm. The deposition process was performed with base pressure below 6.0 × 10^−4^ Pa, while the DC current density was set to 75 A m^−2^, corresponding to a (negative) target potential around 370 V.

### 2.2. Post-Deposition Annealing Treatment

The films were subjected to a post-deposition thermal annealing treatment at atmospheric pressure to promote the formation and growth of the NPs throughout the TiO_2_ matrix, consequently developing an enhanced plasmonic (LSPR) behavior. In a programmable in-air muffle furnace (model LE 6/11/R7 from Nabertherm, Lilienthal, Germany), the thin films were annealed at different temperatures, ranging from 400 to 700 °C [50]. The heating ramp used was 5 °C/min, from room temperature to reach the predetermined annealing temperature, and an isothermal period was maintained for 5 h. Subsequently, the thin films cooled freely inside the furnace until they reached room temperature.

### 2.3. Chemical Composition Analysis

Rutherford Backscattering Spectrometry (RBS) was utilized to determine in-depth chemical concentrations of the as-deposited thin films, using a Van Graaff accelerator (model AN-2500 Type-A from High Voltage Engineering Europe, Amersfoort, Netherlands) in a small chamber with three detectors. Inside the chamber, 2.2 MeV ^4^He^+^ beam impinged on the surface (1 mm^2^) of thin films at an incidence angle of 0° (normal incidence) and 25°. A standard detector was placed at 140°, and two pin-diode detectors were positioned symmetrically to each other at 165° of scattering angle respective to the beam direction. The RBS data were analyzed using the IBA DataFurnace NDF v10.0b software [51,52,53].

### 2.4. Optical Response Measurements

A custom-made optical system was utilized to investigate the optical response of the nanocomposite thin films. The system comprised a tungsten light source, an enclosed thin film holder, and an Ocean Optics spectrometer (model HR4000, Edinburgh, UK), all interconnected by optical fibers, which operated in transmittance mode. The optical measurements were conducted by observing the transmittance spectra within the wavelength range from 300 to 900 nm. The NANOPTICS software (v2022a) [54] was used to analyze the LSPR bands’ parameters (i.e., wavelength and transmittance coordinate at the transmittance minimum, full height, and full width at half height (FWHH)).

### 2.5. Nanoparticles Distribution Analysis by SEM

The morphology of a selected nanocomposite thin film system was examined using Scanning Electron Microscopy (SEM), using an ultra-high resolution Field Emission Gun Scanning Electron Microscope (FEG-SEM) (NOVA 200 Nano SEM, FEI Company, Hillsboro, OR, United States). The SEM micrographs were obtained at an acceleration voltage of 10 kV, using an ultra-high resolution detector (Through-lens detector (TLD)). The SEM micrographs were analyzed using a MATLAB algorithm to determine the NPs distributions (i.e., average Feret diameter, nearest neighbor (N.N.) distance, and aspect ratio (A.R.)).

### 2.6. Thin Film Surface Nano-Morphology by AFM

Atomic Force Microscopy (AFM) analysis was performed using a high-resolution Nano-Observer AFM microscope by CSI Instruments (Les Ulis, France). The AFM microscope was operated in resonant mode using an SPM probe model FORTA (n-type Si; resonance frequency 43–81 kHz; spring constant 0.6–3.7 N/m; tip radium < 10 nm). The AFM height images were scanned at 5 × 5 μm^2^ and 10 × 10 μm^2^, with a resolution of 1024 × 1024 pixels. After performing the AFM scans, the topography profiles were analyzed using the freely available Gwyddion software (v2.59).

### 2.7. Refractive Index Sensitivity Tests

Refractive Index Sensitivity (RIS) was assessed by measuring the optical response of nanocomposite thin films, annealed at 400 and 700 °C, at different surrounding media with various refractive indices (RIs). The surrounding media switched between deionized water (DI water) (η = 1.333 RIU) and a 30% (*w*/*w*) sucrose solution (η = 1.3811 RIU) at room temperature. During the immersion, the transmittance spectra were monitored in real-time for 1 min (per half-cycle) using an Ocean Optics spectrometer (model HR4000, Edinburgh, UK) with an integration time of 4 ms and an average of 500 scans. The NANOPTICS software (v2022a) analyzed the average wavelength peak position of the LSPR band over time and estimated the RIS value. This RIS value was calculated by the ratio of the average wavelength shift to the RI difference of the surrounding medium, according to RIS = (λ_2_ − λ_1_)/Δη. Furthermore, the NANOPTICS software (v2022a) also considered the signal-to-noise ratio (SNR) of the measurements.

## 3. Results

### 3.1. Chemical Composition Analysis

For this work, different sets of thin films were produced: monometallic Au-TiO_2_ and Ag-TiO_2_ and bimetallic AuAg-TiO_2_ thin films. The atomic concentration of the noble metal elements (Au, Ag) dispersed in the TiO_2_ matrix varied as a function of the number of Au and/or Ag pellets placed on the titanium target (Table 1).

In the case of the Au-TiO_2_ thin film system, the noble metal content was found to be 16.7 ± 0.5 at.%. In one of the prepared AuAg-TiO_2_ thin films, it was estimated a noble metal concentration of 8.6 (± 0.5) at.% for Au and 9.1 (±0.5) at.% for Ag. Despite the thin film being prepared with distinct metal pellet areas (8 mm^2^ of Au and 4 mm^2^ of Ag), the Au/Ag atomic ratio was very close to 1. Another bimetallic film, prepared with 4 mm^2^ of Au and 8 mm^2^ of Ag pellet areas, exhibited a metal composition of 6.5 (±0.5) at.% for Au and 11.8 (±0.5) at.% for Ag, resulting in an Au/Ag atomic ratio approaching 2. The distinct Au/Ag atomic ratio results from the difference in sputtering yield between Ag (2.20) and Au (1.65) during the deposition process [55], thus translating into a higher sputtered Ag content compared to the Au for the same pellet area. Finally, regarding the Ag-TiO_2_, the Ag content was found to be 18.2 ± 0.5 at.%.

### 3.2. Optical Response Analysis

**Figure 3:**
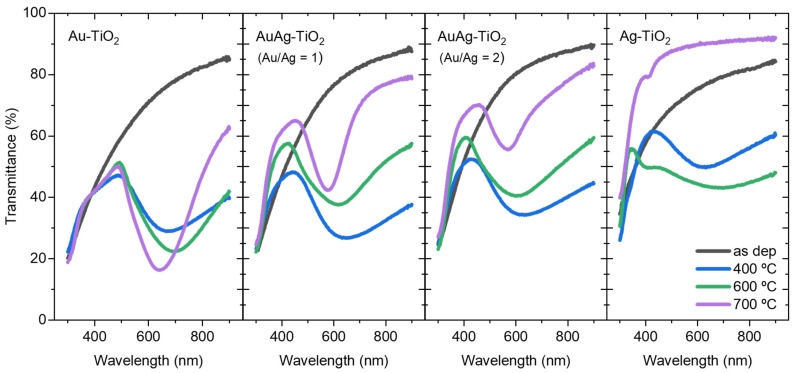
Optical transmittance spectra of the Au-TiO_2_, AuAg-TiO_2_, and Ag-TiO_2_ films with different Au and/or Ag contents and subjected to different annealing temperatures.

The optical transmittance spectra of the nanocomposite thin films were analyzed at various annealing temperatures (400, 600, and 700 °C) to evaluate their relevant plasmonic response, as depicted in Figure 3.

Despite the possible presence of non-crystallized NPs formed during the sputtering process, the transmittance profiles of all thin films exhibited no LSPR response. As Koneti et al. [56] stated, this negligible plasmonic response can be attributed to the limited size of the metallic NPs within the matrix, most likely a few nanometers below the quantum size limit (around 10 nm). In contrast, the annealing treatment in the nanocomposite thin film favors the metallic atoms diffusion within the TiO_2_ matrix and the progressive formation of the NPs. As the annealing temperature increases, the small-size group of the NPs acts as nucleation sites for the crystallization and “normal growth” of the monometallic and/or bimetallic NPs. The coalescence of adjacent NPs and Oswald ripening phenomena may also occur at higher temperatures [57]. As a result, the plasmonic response is enhanced, leading to changes in the LSPR bands’ measurable parameters, as shown in Table 2.

According to the literature, higher annealing temperatures induce the NPs size increase, enhancing the scattering-to-absorption ratio, and consequently, the LSPR band tends to shift to higher wavelengths [5,56,58,59]. In fact, according to the simulations depicted in Figure 1, both the Ag and Au NPs display a reasonable redshift as their size increases. However, it is crucial to note that the calculations presented in Figure 1 are intended solely for qualitative correlation between the LSPR behavior and Ag and Au NP sizes. A quantitative correlation analysis, such as the exact position of extinction maxima (that corresponds to transmittance minima), cannot be performed since the thin film nanomaterial prepared experimentally comprises a wide distribution of NP sizes, revealing it as a more complex system.

Nevertheless, as perceivable from Figure 3 and taking into consideration the calculations in Figure 1, the observed trends are different from what would be expected. All the LSPR bands of Au-containing systems shifted to lower wavelengths (blueshift) when the temperature increased from 600 to 700 °C. The plasmonic behavior of NPs is influenced by the RI of their surrounding medium. Yet, the effective RI of the medium surrounding the NPs might be influenced not only by the host TiO_2_ matrix. The smaller NPs would be fully embedded within the matrix, while the larger ones could be partially exposed. As observed in previous work [60], the TiO_2_ matrix crystallization favors the presence of “grooves” resulting from grain boundary formation. These grooves serve as pathways that facilitate the growth of the NPs towards larger sizes, enabling them to “emerge” on the surface of the thin film. In turn, the TiO_2_ matrix crystallizes into two phases: anatase and rutile, above 300 °C and 600 °C, respectively, with successively higher RIs [61,62]. TiO_2_ not only tunes the LSPR band by modulating the surrounding refractive index, but also constrains the NPs size distribution [30]. Considering that the plasmon decay length of the NP is typically around 30 nm [28,29] and the estimated thickness of the film is approximately 50 nm, the effective RI of the dielectric medium (air plus matrix) will thereby significantly influence the LSPR band position.

In Ag-containing systems, the Ag concentration deeply influences the LSPR band position. As the Ag content in the nanocomposite thin film increases, the LSPR band shifts towards lower wavelengths, considering the same annealing temperature. This LSPR band blueshift can be primarily attributed to the increased contribution of Ag and bimetallic NPs to the overall extinction cross-section. In fact, LSPs in Ag NPs are excited at lower wavelengths than in Au NPs, and hence, the difference in excitation wavelengths between Ag and Au NPs would be predictable [63]. This distinct LSPR behavior between Ag and Au NPs can also be confirmed by the simulations presented in Figure 1. Nevertheless, as aforementioned, the annealing treatment may induce certain instability in the Ag NPs, leading to the formation of unique NP aggregation patterns and, beyond, making them more susceptible to oxidation and degradation. Particularly, in the case of the Ag-TiO_2_ film annealed at 600 °C, the broad LSPR band may indicate the presence of Ag fractal structures on the film’s surface, as already observed in previous works [61,64]. At higher annealing temperatures (700 °C), the Ag NPs may suffer from sublimation [65,66], leading to a vanishing of the plasmonic behavior. Yet, in the bimetallic systems, this behavior seems to be avoided due to the presence of gold, as the occurrence of the LSPR bands confirms (Figure 3). Therefore, as anticipated in the introductory section, incorporating Au in bimetallic systems seems to improve the stability and plasmonic behavior of Ag-containing films. Furthermore, the highly stable host TiO_2_ matrix that is embedding the NPs, will also confer that long time stability to the plasmonic system.

Still focusing on the LSPR band minimum (extinction maximum), it is anticipated that the transmittance coordinate would decrease with increasing annealing temperature. This LSPR band transmittance decrease is indicative of enhanced plasmonic behavior, which arises from the formation of a greater number of NPs with increasing dimensions, resulting from the atoms dissolved within the host matrix and coalescence phenomena [57]. This phenomenon is particularly notable in the monometallic (Au) thin films. On the other hand, the transmittance coordinates of the LSPR bands of Ag-containing films have increased. Unlike the Au-TiO_2_ film, the annealing process in bimetallic films may lead to some Ag sublimation, decreasing the overall noble metal content. Consequently, the transmittance spectra of such films deviate to higher transmittance values.

In addition, other LSPR band parameters evolved with the annealing temperature, namely the full height and the FWHH. Commonly, the LSPR bands tend to become sharper (i.e., the increase of full height) and narrower (i.e., the decrease of FWHH) with an enhanced plasmonic resonance behavior [4,23,50]. Once again, the Au-TiO_2_ thin film system followed this trend [56]. However, the increment of the Ag content in the bimetallic thin films caused a decrease in the full height of the LSPR band, suggesting again that only the bimetallic NPs remain, and the “excess” of Ag probably has sublimated. Consequently, the concentration of noble metal in the films decreases with the annealing treatment, affecting the full height. 

In contrast, the FWHH of the LSPR band decreased in all the plasmonic nanocomposite thin film sets with the annealing treatment, indicating that the dispersion of the NPs size decreases, thus fine-tuning their distribution. For instance, the NP’s sensing capability can be characterized through a figure of merit (FoM) determined by a ratio between the sensitivity and the FWHH of the LSPR band [4,67,68,69]. A high FoM (and sensitivity) is mainly caused by a narrow FWHM [70]. Hence, it can be claimed that the annealing treatment at higher temperatures may improve the sensing ability of the plasmonic nanocomposite thin film.

### 3.3. Refractive Index Sensitivity

The sensing performance of the plasmonic nanocomposite thin films was evaluated by monitoring the LSPR band, by exposing them to different surrounding media with varying refractive index, repeated for a total of five cycles. For the RIS assay, only the plasmonic nanocomposite thin films annealed at 400 °C and 700 °C were considered. The lowest annealing temperature was contemplated since it allows the application of plasmonic films on economically viable substrates such as glass, thereby enabling the development of cost-effective nanotechnology solutions. On the one hand, the highest temperature was used since it gave the narrower bands for all the Au-containing films. The plasmonic sensing ability was characterized based on three parameters: the average wavelength peak position of the LSPR band, SNR, and calculated RIS values, as depicted in Table 3.

Considering the lowest annealing temperature, 400 °C, the sensitivity of the monometallic plasmonic nanocomposite thin films, both the Au-TiO_2_ and Ag-TiO_2_, could not be determined. The LSPR band position of the Au-TiO_2_ thin film, annealed at 400 °C, is 671.41 nm (as mentioned above), which is very close to the wavelength range where the noise contribution of the optical system is significant. In this case, the high noise contribution lowered the SNR, leading to a non-reproducible sensing test. Furthermore, the Ag-TiO_2_ thin film did not demonstrate a consistent plasmonic response during the sensitivity tests.

**Figure 4:**
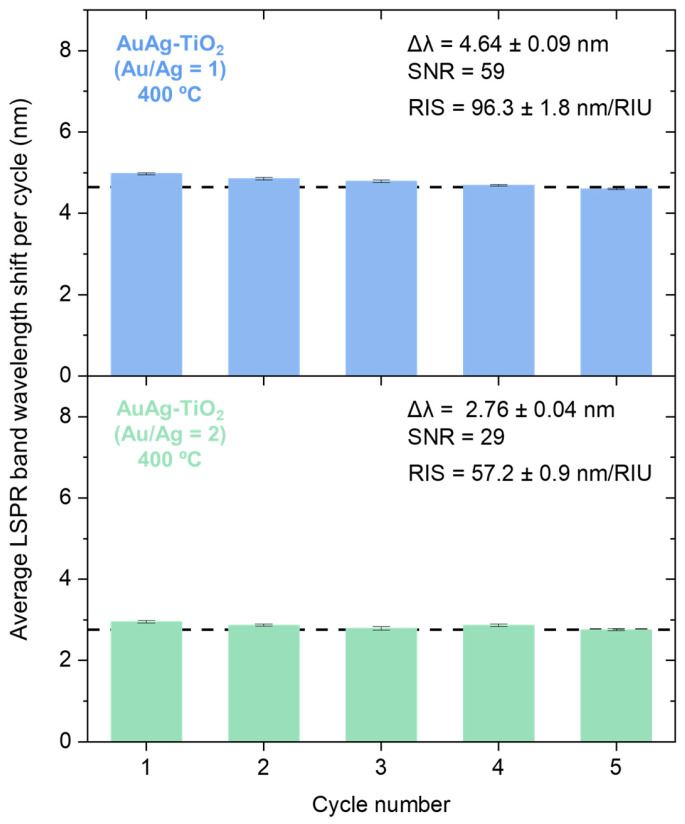
LSPR band minimum wavelength shift of AuAg-TiO_2_ thin films (Au/Ag atomic ratio of 1 and 2), annealed at 400 °C, during the RIS assessment, concerning each cycle. The surrounding media of the plasmonic nanocomposite thin film changed between DI water and sucrose solution of 30% (*w*/*w*) of sucrose.

In turn, the RIS value was successfully determined in the bimetallic systems annealed at 400 °C, by measuring the LSPR band peak position shift in response to different RI, as presented in Figure 4. In the AuAg-TiO_2_ plasmonic nanocomposite thin film, with an Au/Ag atomic ratio of 1, the wavelength peak shifted by approximately 4.64 ± 0.09 nm. However, the wavelength shifts of the AuAg-TiO_2_ thin film, with the highest Ag content, decreased to around 2.76 ± 0.04 nm. As a result, the RIS values were determined at 96.3 ± 1.8 nm/RIU and 57.2 ± 0.9 nm/RIU, with an SNR of 59 and 29, for the two bimetallic systems, respectively. 

**Figure 5:**
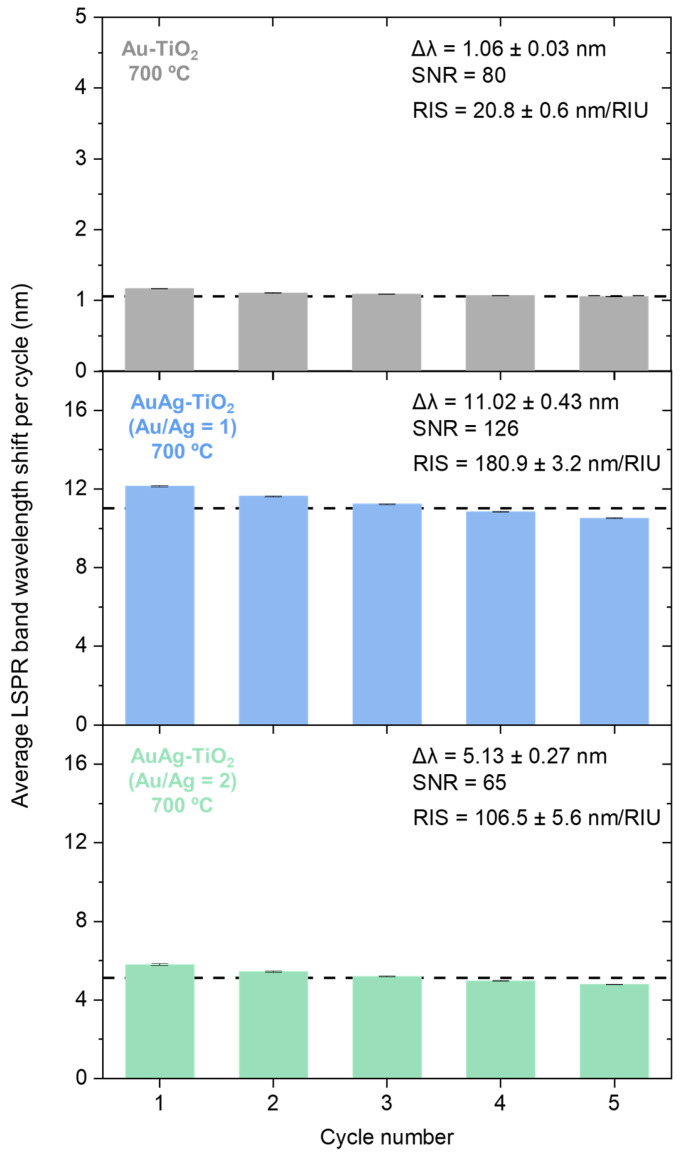
LSPR band minimum wavelength shift of Au-TiO_2_, and AuAg-TiO_2_ thin films (Au/Ag atomic ratio of 1 and 2), annealed at 700 °C, during the RIS assessment, concerning each cycle. The surrounding media of the plasmonic nanocomposite thin film changed between DI water and sucrose solution of 30% (*w*/*w*) of sucrose.

The effect of the thermal annealing treatment induces different nanostructural changes, namely the increase of the size of the NPs, leading to enhanced sensitivity of the nanocomposite thin films, as anticipated by the simulations depicted in Figure 2. Therefore, the sensing response of the Au-TiO_2_ and AuAg-TiO_2_ thin films annealed at the highest annealing temperature is plotted in Figure 5.

Specifically, the Au-TiO_2_ thin film annealed at 700 °C exhibited a sharper and more intense LSPR band positioned at lower wavelengths than 400 °C, enabling accurate RIS determination. In this specific case, the peak position of the LSPR band shifted approximately 1.06 ± 0.03 nm in response to media with different RI, corresponding to an RIS value of 20.8 ± 0.6 nm/RIU, with an SNR of 80. In turn, the AuAg-TiO_2_ thin film, with a lower Ag content, exhibited a more significant shift in the LSPR band of 11.02 ± 0.43 nm, leading to a higher RIS estimation of 180.9 ± 3.2 nm/RIU and SNR of 126. Conversely, the bimetallic system, with an Au/Ag atomic ratio of 2, displayed a more reduced wavelength peak shift of 5.13 ± 0.27 nm, resulting in an RIS value of 106.5 ± 5.6 nm/RIU and SNR of 65.

The combination of specific bimetallic compositions and annealing conditions plays a critical role in optimizing the plasmonic properties and sensitivity of the thin films for sensing applications. Particularly, the inclusion of the Ag content into the Au-TiO_2_ resulted in higher LSPR band shifts, leading to an improved RIS performance. Notably, the bimetallic system with an Au/Ag atomic ratio close to 1 consistently demonstrated the highest RIS performance compared to the others, regardless of the annealing temperature. Reinforcing the previous assumptions, the sensing ability increased at higher annealing temperatures, reaching a notable value of 181 nm/RIU. Compared to other Au/Ag bimetallic nanocomposites, the sensing ability was considerably similar to numerical modeling using a unit approach [71], and higher than thermal embedded Au–Ag bimetallic thin films [72,73]. This specific bimetallic combination facilitated the formation of stable bimetallic NPs in nanocomposite thin films, where the Au NPs contribute to the stability of the Ag. In turn, the Ag seems to confer an enhanced plasmonic effect and, consequently, improves the sensitivity compared to the Au-TiO_2_. However, when the Au/Ag atomic ratio further increases (Au/Ag = 2), the sensitivity of the plasmonic film drops again. This decline can be attributed to the lower amount of Au required to maintain the stability of bimetallic NPs in the film, diminishing the film’s ability to respond to changes in the RI.

### 3.4. Morphological Analysis of High-Sensitive AuAg-TiO_2_ Thin Film

**Figure 6:**
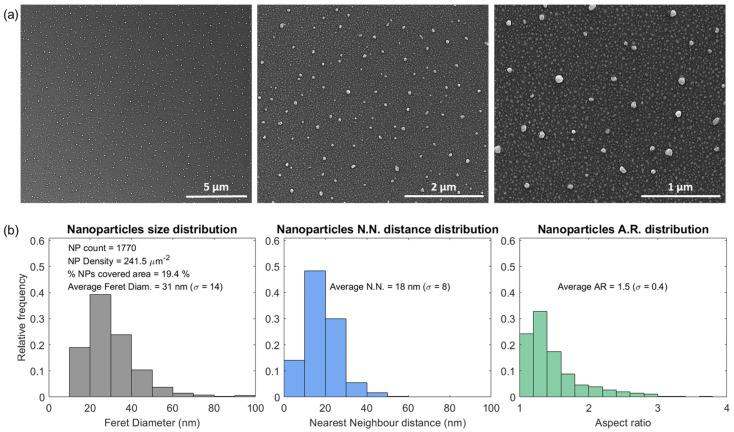
The SEM micrographs of the AuAg-TiO_2_ thin film annealed at 700 °C, at increasing amplifications in (**a**), and the bimetallic NPs size distributions analysis in (**b**), considering the Feret diameter, nearest neighbor distance, and aspect ratio parameters.

**Figure 7:**
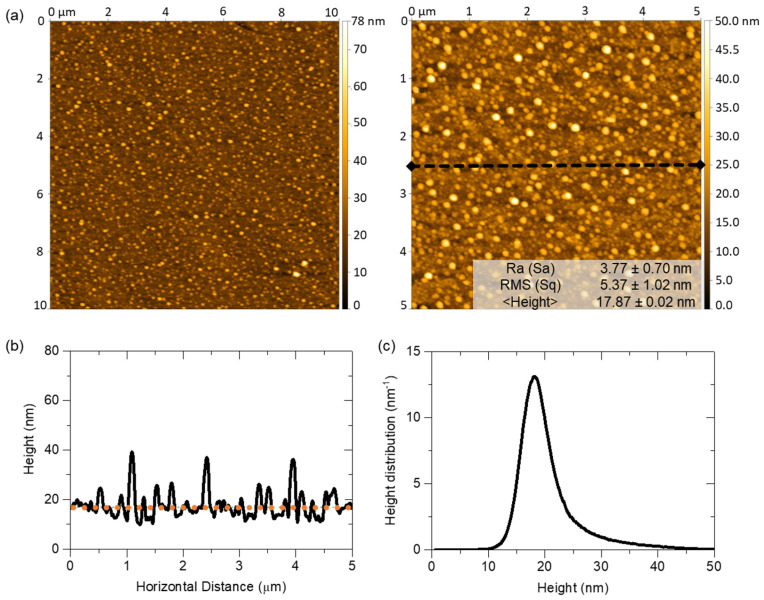
The Morphological analysis of the AuAg-TiO_2_ thin films annealed at 700 °C: (**a**) two-dimensional (2D) AFM height images of 10 × 10 µm^2^ and 5 × 5 µm^2^, with roughness and height parameters. Considering the 2D AFM height image 5 × 5 µm^2^, the topographic profile (delimited in the black dashed line in (**a**)), average height estimation (delimited by orange dotted line), and height distribution profile are represented in (**b**), and (**c**), respectively.

The AuAg-TiO_2_ thin film with an Au/Ag atomic ratio of 1, annealed at 700 °C, was specifically chosen for surface morphological analysis (Figure 6 and Figure 7) based on the earlier optical characterization and its notable sensitivity.

The SEM top-view micrographs of the nanocomposite thin film show a characteristic pattern of roughly spherical NPs, characterized by bright semi-circular spots, surrounded by a darker background that corresponds to the TiO_2_ matrix (Figure 6a). The NPs size distributions of the AuAg-TiO_2_ thin film were analyzed using the SEM top-view micrograph at the highest amplification (at right), plotted in Figure 6b. The metallic NPs covered 19.4% of the total surface area, corresponding to a total countable NP number of 1770 and NP density of 241.5 particles.µm^−2^. The NP size distribution analysis revealed an average NP size (<Feret diameter>) of 31 nm, with a broad distribution of sizes (σ = 14), dispersed with an average distance (<Nearest Neighbor>) of 18 nm (σ = 8). Noteworthy, the NPs with sizes smaller than 10 nm were intentionally disregarded in the analysis owing to their negligible extinction efficiency and plasmonic response, as previously explained in the discussion of Section 3.2. The SEM micrographs showed that most of the NPs are close to spherical, while about 30% are irregularly shaped, resulting in an average aspect ratio of 1.5 nm (σ = 0.4). These slightly elongated NPs (with an aspect ratio > 1) result from the adjacent NPs merging, probably induced by the coalescence phenomena during the annealing treatment [57] and may be associated with enhanced sensitivity exhibited by the plasmonic nanocomposite thin film [4,58].

To complement the morphological analysis, the surface morphology of the AuAg-TiO_2_ thin film was studied using two-dimensional (2D) height images scanned 10 × 10 µm^2^ and 5 × 5 µm^2^ obtained through the AFM analysis, shown in Figure 7. The scanning images enabled the estimation of morphological parameters, topographic profile, and height distribution. 

The 2D AFM height images depict a heterogeneous morphology of the thin film’s surface, characterized by numerous grain-like structures, most likely related to the presence of the NPs near or at the film’s surface (Figure 7a), as predicted by the LSPR band position trends. Moreover, the topographic profile, identified by the black dashed line in Figure 7a, reveals the distinct presence of higher (hills) and lower (valleys) regions. The hills can be attributed to the presence of NPs surrounded by the TiO_2_ matrix, or close to the surface, while the valleys correspond to the host matrix that separates the NPs. From the topography scans of the thin film, the surface mean roughness (Sa) and root mean square roughness (Sq) were estimated to be 3.77 ± 0.70 nm and 5.37 ± 1.02 nm, respectively. The height distribution ranged from 0 to 50 nm; however, the average height was estimated to be 17.87 ± 0.02 nm, as a demarcated orange dotted line in Figure 7b and represented in Figure 7c. Once again, this analysis suggests that the NPs are partially exposed at the film’s surface, highlighting the matrix’s ability to confer robustness while preserving their optical activity for sensing purposes. Both characterization analyses offer complementary information about the surface morphology of the AuAg-TiO_2_ thin film pertinent to forthcoming sensing applications.

## 4. Conclusions

This study aimed to investigate the influence of noble metal (Au, Ag) composition on the sensing capabilities of a plasmonic nanocomposite thin film. Monometallic and bimetallic thin films were produced, including the Au-TiO_2_, AuAg-TiO_2_, and Ag-TiO_2_ thin films. The monometallic thin films exhibited a metal concentration of around 18 at.%. In turn, the bimetallic film system with an Au/Ag atomic ratio of 1 was achieved by adjusting the area covered by the Au twice the Ag pellets area on the target. Conversely, when the dimensions of the Au pellets were halved compared to the Ag pellets on the target, the Au/Ag atomic ratio approached 2.

Among these films, the AuAg-TiO_2_ film with an Au/Ag atomic ratio of 1 exhibited a distinct optical response characterized by a well-defined LSPR band, specifically at the highest annealing temperature (700 °C). Furthermore, it demonstrated higher sensing capability, displaying a remarkable RIS value, exceeding other plasmonic film systems. The film’s surface morphology characterization revealed a relevant metallic surface coverage area very close to 20%. Notably, the surface topography exhibited distinct features, where the NPs and their immediate surrounding matrix were observed as hills, while the TiO_2_ matrix itself appeared as valleys. In conclusion, taking theoretical simulations as a starting point, the findings confirm that it is possible to optimize the extinction efficiency and plasmonic sensitivity of bimetallic systems composed of the Au-Ag NPs embedded in a host TiO_2_ matrix. Notably, the film with an Au/Ag atomic ratio of 1 was revealed as a highly promising transducer platform for the LSPR-based sensors.

## Figures and Tables

**Table 1:** The atomic concentration of the Au and Ag in nanocomposite Au-TiO_2_, AuAg-TiO_2_, and Ag-TiO_2_ thin film systems.

Plasmonic System	Pellet Dimension		Pellet Area (mm^2^)		Atomic Percentage (at.%)		Atomic Concentration Ratio (Au/Ag)
	Au	Ag	Au	Ag	Au (±0.5)	Ag (±0.5)	
Au-TiO_2_	¾	-	12	-	16.7	-	-
AuAg-TiO_2_	½	¼	8	4	8.6	9.1	1
	¼	½	4	8	6.5	11.8	2
Ag-TiO_2_	-	¾	-	12	-	18.2	-

**Table 2:** LSPR band parameters of Au-TiO_2_, AuAg-TiO_2_ (Au/Ag atomic ratio of 1 and 2), and Ag-TiO_2_ films subjected to different annealing temperatures.

Plasmonic System	Annealing Temperature	LSPR Band Parameters			
		Wavelength (nm)	Transmittance (%)	Full Height (p.p.)	FWHH (nm)
Au-TiO_2_	400 °C	671.93	28.93	18.27	292.09
	600 °C	693.19	22.90	28.48	281.76
	700 °C	639.41	16.07	33.96	190.95
AuAg-TiO_2_ Au/Ag = 1	400 °C	641.66	26.69	21.56	367.39
	600 °C	613.98	37.97	19.64	257.22
	700 °C	574.89	42.98	22.04	124.66
AuAg-TiO_2_ Au/Ag = 2	400 °C	626.17	34.22	18.17	358.01
	600 °C	602.41	40.74	18.76	285.49
	700 °C	568.65	56.28	13.89	130.55
Ag-TiO_2_	400 °C	627.46	49.86	11.63	272.24
	600 °C	*-*	*-*	*-*	*-*
	700 °C	*-*	*-*	*-*	*-*

**Table 3:** The optical response analysis of Au-TiO_2_, AuAg-TiO_2_ (Au/Ag atomic ratio of 1 and 2), and Ag-TiO_2_ films, annealed at 400 °C and 700 °C, to the sensitivity assay, considering the LSPR band wavelength shift, SNR and RIS (nm/RIU) values.

Plasmonic System	Annealing Temperature	Wavelength Shift (nm)	SNR	RIS (nm/RIU)
Au-TiO_2_	400 °C	-	-	-
	700 °C	1.06 ± 0.03	80	20.8 ± 0.6
AuAg-TiO_2_ Au/Ag = 1	400 °C	4.64 ± 0.09	59	96.3 ± 1.8
	700 °C	11.02 ± 0.43	126	180.9 ± 3.2
AuAg-TiO_2_ Au/Ag = 2	400 °C	2.76 ± 0.04	29	57.2 ± 0.9
	700 °C	5.13 ± 0.27	65	106.5 ± 5.6
Ag-TiO_2_	400 °C	-	-	-
	700 °C	-	-	-

## Data Availability

The data presented in this study are available on request from the corresponding author.
